# When Time and Numerosity Interfere: The Longer the More, and the More the Longer

**DOI:** 10.1371/journal.pone.0041496

**Published:** 2012-07-20

**Authors:** Amir Homayoun Javadi, Clarisse Aichelburg

**Affiliations:** 1 Section of Systems Neuroscience, Technische Universität Dresden, Dresden, Germany; 2 Division of Psychology and Language Sciences, University College London, London, United Kingdom; National University of Singapore, Singapore

## Abstract

There is strong evidence that magnitudes in different dimensions can interfere. A majority of previous studies on the interaction of temporal magnitudes on numerosity showed no interfering effect, while many studies have reported the interference of numerosity on judgement of temporal magnitudes. We speculated that this one-way interference is confounded by the magnitudes used in the studies. We used a methodology that allowed us to study this interaction reciprocally. Moreover, we selected magnitudes for two dimensions that enabled us to detect their interfering effects. Participants had to either judge which of two successive sets of items was more numerous (numerosity judgement task), or which set of items was presented longer (duration judgement task). We hypothesised that a longer presentation of a set will be judged as being more numerous, and vice versa, a more numerous set will be judged as being presented longer. Results confirmed our hypothesis. A positive correlation between duration of presentation and judged numerosity as well as a positive correlation between the number of items and judged duration of presentation was found. This observation supports the idea that duration and numerosity judgements are not completely independent and implies the existence of (partly) generalised and abstract components in the magnitude representations.

## Introduction

A magnitude is the size or extent of something, such as quantity, length, duration, speed, brightness, weight and position. Magnitudes in different dimensions, such as time or space, are an integral part of our existence. In some circumstances, these interconnections interfere and can lead to a misperception of one dimension or another (see below).

There are many studies on the interaction of numerosity or time with other dimensions, as well as with each other. The majority of the developmental research [1]–[4], as well as research on adults (see below), have confirmed the existence of such an interaction showing that magnitude judgements in different dimensions are sometimes affected by other dimensions. Stavy and Tirosh [1] suggested an intuitive ‘more A-more B’ mapping between different dimensions, e.g. the bigger the trains are the faster they are perceived and etc. In a more comprehensive way, Walsh [5], [6] proposed a theory of magnitude (ATOM), by which he suggested that commonalities between different dimensions such as time, space, number, size and other magnitudes are found in a common brain area (parietal cortex specifically) (for a revision see [7]).

Droit-Volet, Clement and Fayol [8] in a study on children (5 and 8 years old) using time and numerical bisection tasks showed that while numbers interfered with the 5-year-olds' temporal performance, duration did not interfere with numerical discrimination in any age group. Participants were trained to classify the presented stimuli into one of two sequence types, namely ‘short-few’ (2 items, 2 s) and ‘long-many’ (8 items, 8 s) types. Subsequently, the participants were tested with ‘time-varying’ and ‘number-varying’ trials. In time-varying trials, the number of the items in each sequence was kept constant and the duration varied, while in number-varying trials, the duration was kept constant and the number of items varied. In the first experiment participants were asked to ignore the numerosity of the sequences and base their decision solely on the duration of the sequence, while conversely, for the second sequence, they were asked to ignore the duration of the sequences and base their decision on the numerosity of the sequences. Their results clearly showed that different durations did not interfere with the judgement of the number of items in each sequence.

Dormal, Seron and Pesenti [9] used a stroop task in order to investigate the possibility of a common mechanism for duration and numerosity processing. Participants took part in two separate studies; half of them were assigned to the numerosity judgement task and the other half to the duration judgement task. They showed two series of flashing circles with well-controlled on and off durations. Participants were asked to select the more numerous set (numerosity judgement task) or the longer one (duration judgement task). Their results convincingly showed that the difficulty of the two tasks was matched, and the main results were not due to a difference in overall complexity of the tasks. They, however, did not reject the possibility of the participants using a counting strategy in the numerosity judgement task. Similar to Droit-Volet et al. [8], their results showed a unidirectional interference between numerical and temporal cues: numerical cues interfered with duration judgement, but temporal cues did not interfere with numerosity judgement.

Xuan, Zhang, He and Chen [10] in a more comprehensive study, investigated the one-way interaction of time (duration of presentation) and magnitudes in nontemporal dimensions, namely number of dots, size of open squares, luminance of solid squares and numerical value of digits, using a Stroop-like task. They showed sets of stimuli comprised of 2 or 3 items in three different tasks and asked the participants to judge the duration of presentation of the stimuli (1^st^ task), screens with fixation cross (2^nd^ task) or masks (3^rd^ task). Confirming previous findings [8], [9], their results showed that stimuli with larger magnitudes were judged to be presented longer (measured from error rates). They, however, did not investigate the possible interfering effect of time on the judgment of nontemporal magnitudes.

Similar results have been achieved using symbolic numerosity. Roitman, Brannon, Andrews and Platt [11] showed that numerical sensitivity is finer than temporal sensitivity. They argued that this difference suggests a differential salience of time and number. Oliveri, Vicario and Salerno [12] showed that symbolic numbers can bias a temporal duration judgement. Using a time estimation task, it was found that low digits (i.e. digit 1) lead to an underestimation, whereas high digits (i.e. digit 9) lead to an overestimation of perceived duration, when compared to a fixed stimulus cued with number 5. Oliveri et al. [12] interpreted their results in favour of “a functional interaction between number magnitude processing and time estimation”. Cappelletti, Freeman and Ciplottie [13] compared the performance of a patient with right hemisphere lesion with the performance of a matched control group in a series of tasks in order to study the judgement and interaction of magnitudes in different dimensions (time, numerosity and space). Their results showed a unidirectional interaction between numbers and time in both the patient and the control group. They showed that time was perceived as shorter when cued with small numbers and perceived as longer when cued with larger numbers.

In all previous studies, a unidirectional interaction between numerosity and duration processing has been found (low and high numerosity led to shorter and longer perception of duration, respectively), and it has been shown that the temporal dimension does not interfere with numerosity. We speculated that these findings are confounded by the selected magnitudes in the two numerosity and temporal dimensions.

Furthermore, the numerosity naming capabilities in adults have been shown to experience a sudden drop (increased response time) for sets containing more than 4 items [14], [15]. The increase in response time correlated with the number of items in the set, which suggests counting and estimating strategies. Therefore, two separate processes are proposed: ‘subitizing’ for small numerosities [16] and an analogue magnitude system for larger sets [17]–[19].

Additionally, it has been suggested that “explicit judgements on numerosity are frequent, whereas judgements on duration are generally made implicitly and prospectively rather than retrospectively” [9]. Moreover, it has been shown that numerosity sensitivity is more salient compared to temporal sensitivity [11]. Therefore, numerosities slightly higher than 3–4 items might still benefit from high accuracy of estimation; thus, they are processed more explicitly compared to temporal judgements that suffer from lack of discrete enumeration. All the sets used in previously mentioned studies on temporal and numerosity interference contained between 1 and 9 items [9]–[11]. We speculated that the interference of the temporal dimension on the processing of numerosity was not strong enough to shift the psychophysical curves to achieve a measurable effect, due to imbalance between magnitudes in the two temporal and numerosity dimensions. In order to study the interference of two dimensions, it is necessary to calibrate the quantities of the dimensions. We used short durations of presentations (53–106 ms) and a high number of items (28–40) in this study to enable implicit measurements in both temporal and numerosity dimensions. Furthermore, we used a methodology that allowed us to investigate this interaction reciprocally rather than only looking at the unidirectional interaction of two dimensions commonly employed in previous research.

We aimed to investigate whether a set of items presented for a longer duration is judged as being more numerous, and vice versa, i.e. whether a set containing more items is judged as being presented longer. Participants were presented with two consecutive sets and were asked to select the set with the higher number of items (numerosity judgement experiment) or to select the set with the longer duration of presentation (duration judgement experiment). To study the effect of duration of presentation on numerosity judgement in the numerosity judgement experiment, in some of the trials the two sets were presented with the same number of items but with different durations, which was unknown to the participants. Similarly, to study the effect of numerosity on time judgement in the duration judgement experiment, in some of the trials the two sets were presented with the same duration but differing in the number of items, also unknown to the participants. Additionally, we ran a control experiment to investigate the contribution of total occupied area by the items in each set to the interference of numerosity and duration judgement.

Based on previous findings, intuitive ‘more A-more B’ mapping [1] and ATOM [5], [6], we expected to see a positive correlation between duration of presentation and judged numerosity (in numerosity judgement experiment) and a positive correlation between the number of items and judged duration of presentation (in duration judgement experiment).

## Methods

### Participants

Thirty-nine (25 females, 18–24 years old) participants took part in three experiments: numerosity judgement experiment (*n* = 12), duration judgement experiment (*n* = 12) and control experiment (*n* = 15). All the participants were healthy with no history of neurological or psychiatric disorder, had normal or corrected-to-normal vision and remained naive to the purpose of the study. They were right-handed yielding a laterality quotient of at least +50 on the Edinburgh Handedness Inventor [20]. All participants gave their written informed consent in accordance with the Declaration of Helsinki and the guidelines approved by the ethical committee of University College London (UCL).

### Apparatus

Experiments were run on desktop computers with a 17-inch CRT monitor and 75 Hz refresh rate with the resolution 1024×768 pixels. The monitor was 53 cm from participants' eyes. Stimuli presentation and the recording of response time were achieved using MATLAB (v7.5; MathWorks Company) and the Psychtoolbox v3 [21], [22]. Data analyses were performed using SPSS (v17.0; LEAD Technologies, Inc.). Responses were made on a conventional computer keyboard using index and middle fingers of the right hand.

### Stimuli

Stimuli were sets of items composed of the image of a synthetic ball copied in random locations within a 25.32×19.12 visual degrees virtual rectangle at the centre of the monitor on a black background. The items were a solid yellow sphere with a mild shading created by 3DS Max (Autodesk). The size of the items was 1.61×1.61 visual degrees (standard item) in the numerosity and duration judgement experiment, but varied in the control experiment (see below). The centre of no two items could be allocated closer than 5/2 of their radius (0.8 visual degrees).

### Design

**Figure 1:**
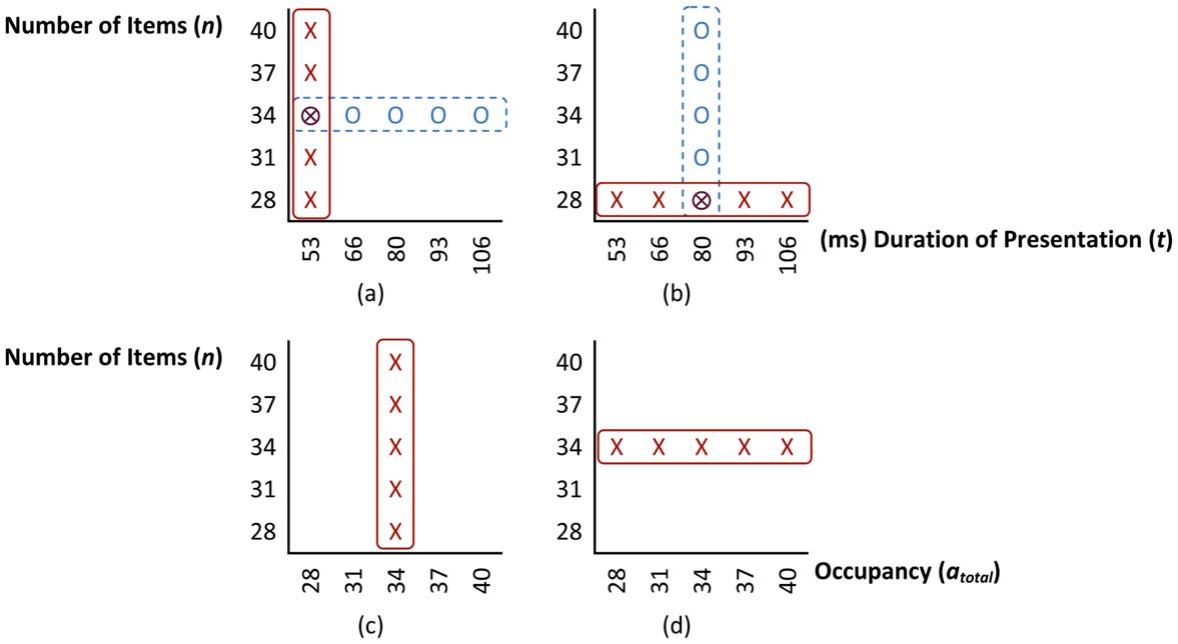
Combination of the numerosity and duration of presentation in each experiment. The number of items of a set and duration of the presentation of a set in (a) numerosity judgement experiment, (b) duration judgement experiment, (c) numerosity judgement phase and (d) occupancy judgement phase in the control experiment. 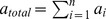 represents total occupied area scaled to the area of one standard item. Symbols in the solid and dashed bars refer to ‘veridical’ and ‘phantom’ conditions, respectively (refer to the text).

The study adopted a within subject design with each subject participating in one experiment: numerosity or duration judgement experiment or control experiment. In the *numerosity judgement experiment*, subjects had to judge which one of the two successive sets is more numerous, whereas in the *duration judgement experiment* participants had to decide which set was presented longer. Within each session, two independent variables, namely the duration of presentation of each set (*t_1_* and *t_2_*) and the number of items in each set (*n_1_* and *n_2_*), were modified. Trials in each session were either ‘veridical’ or ‘phantom’. *Veridical trials* were the trials in which judgement was done based on the varying independent variable known to the participants, i.e. *t_1_* = *t_2_* and *n_1_*≠*n_2_* for numerosity judgement experiment and *t_1_*≠*t_2_* and *n_1_* = *n_2_* for duration judgement experiment. *Phantom trials*, on the other hand, were the trials in which unknown to the participants the values of the judged independent variable was identical for the two sets and variation happened in the interfering dimension, i.e. *t_1_*≠*t_2_* and *n_1_* = *n_2_* for numerosity judgement experiment and *t_1_* = *t_2_* and *n_1_*≠*n_2_* for duration judgement experiment. Figure 1(a, b) show different combinations of *n* and *t* in the two experiments (*n *∈ {28, 31, 34, 37, 40}, *t *∈ {53 ms, 66 ms, 80 ms, 93 ms, 106 ms}).

*Control experiment* composed of two phases with random order: numerosity and occupancy judgement phases. In the numerosity judgement phase participants were asked to judge which one of the two sets contained more items, whereas in the occupancy judgement phase they were asked to judge items in which set occupied more area. In the numerosity judgement phase the number of items changed (*n *∈ {28, 31, 34, 37, 40}) while the total occupied area by the items was kept constant (*a_total,1_* = *a_total,2_* = 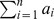 = 34, *a_i _*∈ [0.70..1.42], in which *a_i_* is the scaling factor for item *i* ^th^). The size of each of the items was pseudo-randomly selected (*a_i_*) to remove the possibility of judging the numerosity based on the size of one single item. In the occupancy judgement phase the total occupied area by the items varied (*a_total_* = 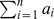
∈ {28, 31, 34, 37, 40}) while the number of items was kept constant (*n_1_* = *n_2_* = 34), Figure 1(c, d) shows the combination of *n* and *a_total_* for the two phases of the control experiment.

### Test Procedure

*Numerosity and duration judgement experiments*; Participants were randomly assigned to either experiment. Each session was composed of eight blocks of 80 trials (8 repetitions per absolute value of difference level |*n_2_*−*n_1_*| and |*t_2_*−*t_1_*|, see below), resulting in 320 veridical and 320 phantom trials in total. Although the trials in which both *n_2_*−*n_1_* = 0 and *t_2_*−*t_1_* = 0 were similar in between veridical and phantom trials, we included separate trials for the two types of trials to keep the number of samples in all conditions equal.

**Figure 2:**
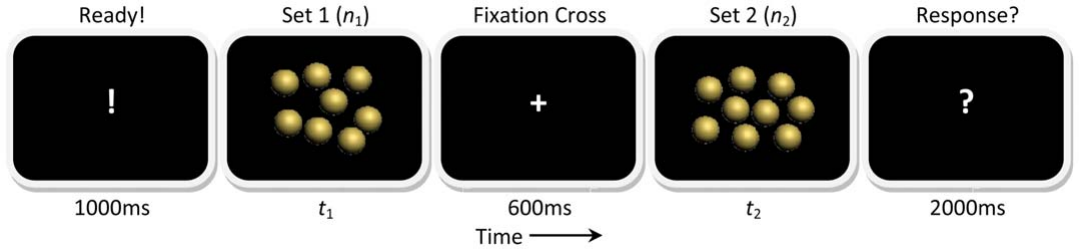
Procedure of a trial. The number of items in this figure is only for illustration, see Figure 1 for details of magnitudes used.

The procedure of one trial is shown in Figure 2. The presentation variables (*n_1_*, *n_2_*, *t_1_* and *t_2_*) were randomly selected from combinations shown in Figure 1(a, b).

After each block feedback was given based on the participant's performance on the veridical trials. Participants were instructed to response as accurately and as quickly as possible within the response period. Participants were also asked to keep their gazing point at the centre of the monitor at all times.

*Control experiment*; The control experiment was ran after the numerosity and duration judgement experiments. It was composed of two phases of eight blocks of 40 trials (8 repetitions per absolute value of difference level |*n_2_*−*n_1_*| and |*a_total,2_−a_total,1_*|, see below), resulting in 320 veridical trails in total for each phase. The presentation variables (*n_1_*, *n_2_*, *a_total,1_* and *a_total,2_*) were randomly selected from combinations shown in Figure 1(c, d). The duration of presentation of each set was 80 ms. The rest of the procedure was identical to that of the numerosity and duration judgement experiments (above).

### Statistical Analysis

Trials with response time lower than 100 ms were excluded. For the numerosity and duration judgement experiments separate one-way repeated measures of analysis of variances (ANOVA) were run for each condition (veridical/phantom) with the percentage of response towards the first set as the dependent variable. For the veridical condition, the independent variable was the difference level of the judged dimension between the two sets, i.e. *k* = *n_2_*−*n_1_* for the numerosity judgement task and *k* = *t_2_*−*t_1_* for the duration judgement task. For the phantom condition, on the other hand, the independent variable was the difference level of the interfering dimension between the two sets, i.e. *k* = *t_2_*−*t_1_* for the numerosity judgement task and *k* = *n_2_*−*n_1_* for the duration judgement task. For the control experiment, separate one-way repeated measure ANOVAs were run for each phase (numerosity/occupancy judgement phases) with the percentage of response towards the first set as the dependent variable and difference level (*k* = *n_2_*−*n_1_* in numerosity judgement phase and *k* = *a_total,2_−a_total,1_* in occupancy judgment phase) as the independent factor. Consequently the independent variable had nine levels, *k* = [−4 .. +4]. Negative values show the first set contained more items, was presented longer or occupied more area. Post-hoc two-tailed one sample *t*-tests were run to compare the ultimate levels (*k* = −4 and *k* = +4) with the zero level (*k* = 0). In order to correct for multiple comparisons, we considered *p* values less than 0.025 as significant.

The response time was also analysed using a 2×9 repeated measures ANOVA with condition (veridical/phantom in numerosity and duration judgement experiments and numerosity/occupancy in control experiment) and different levels of presentation (nine levels) as independent factors.

Two-tailed Pearson correlation analysis was used to study the relation between nine levels of independent factors and dependent factors (percentage of response towards the first set and response time).

## Results

### Numerosity Judgement Task

To investigate the effect of numerosity (veridical trials, *t_1_* = *t_2_*), we ran a one-way repeated measures ANOVA on the numerosity difference of the two sets (nine levels) as independent factor and percentage response towards the first set as dependent factor. This test showed a significant main effect of difference level (*F*(8, 88) = 105.43, *p*<0.001). Bonferroni corrected post-hoc one sample *t*-tests were conducted to look at difference between extreme levels with level zero. These comparisons showed a highly significant difference between level −4 and 0, *t*(11) = 7.75, *p*<0.001, and between level +4 and 0, *t*(11) = 8.57, *p*<0.001.

**Figure 3:**
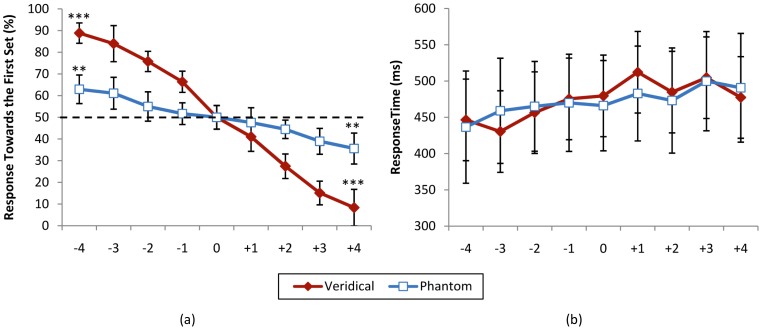
Psychometric function for the numerosity judgement task. (a) Percentage response towards the first set and (b) response time for veridical (*n_1_*≠*n_2_*, *t_1_* = *t_2_*) and phantom (*n_1_* = *n_2_*, *t_1_*≠*t_2_*) conditions. Horizontal axis shows different levels of comparison (*n_2_*−*n_1_* for veridical and *t_2_*−*t_1_* for phantom trials). *** *p*<0.001, ** *p*<0.005. Error bars reflect one standard deviation.

Numerosity judgement in phantom trials (*n_1_* = *n_2_*) was also subjected to a one-way repeated measures ANOVA, with presentation duration difference (nine levels) as independent factor and percentage response towards the first set as dependent factor. This test showed a significant main effect of difference level (*F*(8, 88) = 11.651, *p*<0.001). Post-hoc one sample *t*-tests were conducted to look at difference between extreme levels with level zero. These comparisons showed a significant difference between level −4 and 0, *t*(11) = 4.19, *p* = 0.001, and between level +4 and 0, *t*(11) = 4.43, *p* = 0.001. Figure 3(a) shows the percentage response towards the first set for veridical and phantom conditions for different levels of comparisons.

Pearson correlation analysis showed a significant relation between the nine levels of the independent factor of numerosity and percentage of response towards the first set for veridical trials (*r*(106) = 0.925, *p*<0.001), as well as a significant relation between the nine levels of the independent factor of duration and percentage of response towards the first set for phantom trials (*r*(106) = 0.538, *p*<0.001).

Response times were also subjected to a 2×9 repeated measures ANOVA with condition (veridical/phantom) and nine levels of difference of number of presented items (for veridical trials) or difference of duration of presentation (for phantom trials) as independent factors. This test showed a non-significant main effect of condition (*F*(1, 11) = 0.080, *p* = 0.782), non-significant main effect of difference level (*F*(8, 88) = 1.711, *p* = 0.107) and non-significant effect of interaction (*F*(8, 88) = 1.165, *p* = 0.329). Figure 3(b) shows the response times for veridical and phantom conditions for different levels of comparisons.

Pearson correlation analysis showed a non-significant relation between reaction times and the nine levels of the independent factor of numerosity and duration of presentation (*r*(106) = 0.130, *p* = 0.184).

### Duration Judgement Task

To investigate the effect of duration of presentation of the two sets in duration judgement (veridical trials, *n_1_* = *n_2_*), we ran a one-way ANOVA on the presentation duration difference of the two sets (nine levels) as independent factor and percentage response towards the first set as dependent factor. This test showed a significant main effect of difference level (*F*(8, 88) = 77.036, *p*<0.001). Post-hoc one sample *t*-tests were conducted to look at difference between extreme levels with level zero. These comparisons showed a significant difference between level −4 and 0, *t*(11) = 7.39, *p*<0.001, and between level +4 and 0, *t*(11) = 7.01, *p*<0.001.

**Figure 4:**
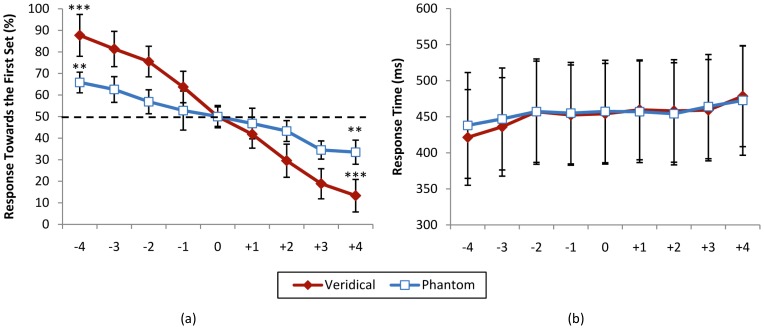
Psychometric function for the duration judgement task. (a) Percentage response towards the first set and (b) response time for veridical (*t_1_*≠*t_2_*, *n_1_* = *n_2_*) and phantom (*t_1_* = *t_2_*, *n_1_*≠*n_2_*) conditions. Horizontal axis shows different levels of comparison (*t_2_*−*t_1_* for veridical and *n_2_*−*n_1_* for phantom trials). *** *p*<0.001, ** *p*<0.005. Error bars reflect one standard deviation.

Duration judgement in the phantom trials (*t_1_* = *t_2_*) was also subjected to a one-way ANOVA, with difference of number of items in the two sets (nine levels) as independent factor and percentage response towards the first set as dependent factor. This test showed a significant main effect of difference level (*F*(8, 88) = 39.433, *p*<0.001). Bonferroni corrected post-hoc one sample *t*-tests were conducted to look at difference between extreme levels with level zero. These comparisons showed highly significant difference between level -4 and 0, *t*(11) = 4.20, *p* = 0.001, and between level +4 and 0, *t*(11) = 4.39, *p*<0.001. Figure 4(a) shows the percentage response towards the first set for veridical and phantom conditions for different levels of comparisons.

Pearson correlation analysis showed a significant relation between the nine levels of the independent factor of numerosity and percentage of response towards the first set for veridical trials (*r*(106) = 0.862, *p*<0.001), as well as a significant relation between the nine levels of the independent factor of duration and percentage of response towards the first set for phantom trials (*r*(106) = 0.707, *p*<0.001).

Response times were also subjected to a 2×9 repeated measures ANOVA with condition (veridical/phantom) and nine levels of difference of duration of presentation (for veridical trials) or difference of number of presented items (for phantom trials) as independent factors. This test showed a non-significant main effect of condition (*F*(1, 11) = 0.600, *p* = 0.455), significant main effect of difference level (*F*(8, 88) = 1.381, *p* = 0.216) and non-significant effect of interaction (*F*(8, 88) = 1.132, *p* = 0.350). Figure 4(b) shows the response times for veridical and phantom conditions for different levels of comparisons.

Pearson correlation analysis showed a non-significant relation between reaction times and the nine levels of the independent factor of numerosity and duration of presentation (*r*(106) = 0.101, *p* = 0.303).

Post-study interviews showed that none of the participants noticed the variation of the other dimension throughout the experiment.

### Control Experiment

To investigate the effect of duration of presentation of the two sets in duration judgement (veridical trials, *n_1_* = *n_2_*).

*Numerosity judgement phase*; we ran a one-way ANOVA on the numerosity difference of the two sets (nine levels) as independent factor and percentage response towards the first set as dependent factor. This test showed a significant main effect of difference level (*F*(8, 112) = 3.61, *p*<0.001). Post-hoc one sample *t*-tests were conducted to look at the difference between extreme levels with level zero. These comparisons showed a significant difference between level −4 and 0, *t*(14) = 3.78, *p* = 0.003, and between level +4 and 0, *t*(14) = 3.89, *p* = 0.002.

Pearson correlation analysis showed a significant relation between the nine levels of the independent factor of numerosity and percentage of response towards the first set (*r*(133) = 0.30, *p*<0.001).

*Occupancy judgement phase;* similarly, we ran a one-way ANOVA on the occupancy difference of the two sets (nine levels) as independent factor and percentage response towards the first set as dependent factor. This test also showed a significant main effect of difference level (*F*(8, 112) = 2.36, *p* = 0.02). Post-hoc one sample *t*-tests were conducted to look at difference between extreme levels with level zero. These comparisons showed a non-significant difference between level −4 and 0, *t*(14) = 2.31, *p* = 0.03, but a significant difference between level +4 and 0, *t*(14) = 2.55, *p* = 0.02.

Pearson correlation analysis showed a significant relation between the nine levels of the independent factor of numerosity and percentage of response towards the first set (*r*(133) = 0.20, *p* = 0.02).

**Figure 5:**
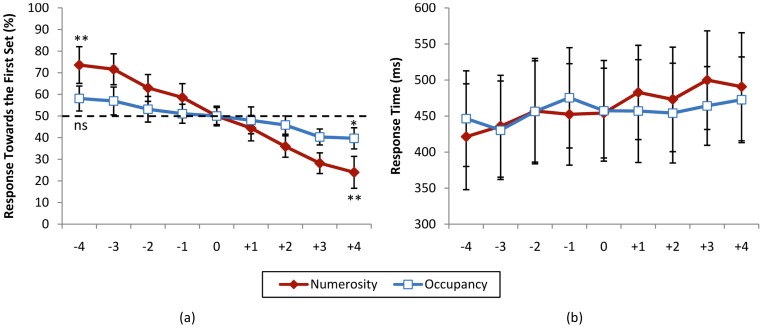
Psychometric function for the control experiment. (a) Percentage response towards the first set and (b) response time for numerosity (*n_1_*≠*n_2_*, *a_total,1_* = *a_total,2_*) and occupancy (*n_1_* = *n_2_*, *a_total,1_*≠*a_total,2_*) judgement phases. Horizontal axis shows different levels of comparison (*n_2_*−*n_1_* for numerosity and *a_total,2_*−*a_total,1_* for occupancy judgement phase). ** *p*<0.005, * *p*<0.025, ns not significant. Error bars reflect one standard deviation.

Figure 5(a) shows the percentage response towards the first set for veridical and phantom conditions for different levels of comparisons.

Response times were also subjected to a 2×9 repeated measures ANOVA with condition (numerosity/occupancy judgement phase) and nine levels of difference of duration of presentation as independent factors. This test showed a non-significant main effect of condition (*F*(1, 14) = 0.82, *p* = 0.38), significant main effect of difference level (*F*(8, 112) = 1.22, *p* = 0.29) and non-significant effect of interaction (*F*(8, 112) = 0.83, *p* = 0.57). Figure 5(b) shows the response times for numerosity and occupancy phases for different levels of comparisons.

Pearson correlation analysis showed a non-significant relation between reaction times and the nine levels of the independent factor of numerosity and duration of presentation (*r*(133) = 0.11, *p* = 0.18).

## Discussion

The interaction of duration and numerosity judgement was investigated in the present study. The research question concerned whether a set of items presented for a longer duration would be judged as being more numerous, and whether a set containing more items would be judged as being presented longer. We hypothesised that judgement of duration and number correlates, the more numerous a set, the longer it is judged and vice versa. In two experiments, we investigated the interference of numerosity and duration judgement.

The hypotheses were confirmed by the results. Confirming previous literature, a significant positive correlation between the number of items and judged duration of presentation (in the duration judgement experiment) was found. Moreover, contrary to previous literature, a significant positive correlation between duration of presentation and judged numerosity (in the numerosity judgement experiment) was found.

The size of items was kept constant in the numerosity and duration judgement experiments, resulting in higher occupancy of the sets with higher numerosity and less occupancy with lower numerosity. Therefore, the results could be confounded by the total occupancy of the sets. In a control experiment we investigated whether participants were able to correctly judge the difference between total occupied areas of the two sets of items and compare it with their ability to judge the numerosity of the two sets. The results of this experiment showed that although participants were partly capable of differentiating between occupancy of the two sets (non-significant in one extreme and significant in another), judgement of numerosity was much easier, Figure 5. Hence, we argue that although occupancy might also contribute in the reported interference between numerosity and duration judgement, it is the numerosity that plays the major role in this interference.

There is an ongoing debate on the mechanisms underlying processing magnitudes in different dimensions. Brain imaging, brain stimulation and lesion studies as well as behavioural studies have been undertaken to find brain regions involved in this process. Several brain regions are suggested to be involved in numerosity and temporal processing. There is strong evidence of bilateral activation of intraparietal sulci (IPS) and fronto-parietal in numerical cognition [23]–[33]. Studies on duration perception showed a contribution of a more diverse network of brain areas. It has been shown that the basal ganglia, cerebellum and more importantly the parietal and frontal cortices and supplementary motor areas play a key role in duration perception [13], [34]–[44] (for a review see [45] and meta-analysis see [46]).

As a result of these studies and others concerning the perception of space, Walsh [5], [6] proposed in a theory of magnitude (ATOM) the parietal cortex to be the common brain area involved in perception of time, space, number, size, speed and other magnitudes (for a revision see [7]). ATOM revolves primarily around the role of parietal cortex (as the major area for sensory integration and object manipulations), needed for active interactions with the environment in order to acquire knowledge. This theorem, however, does not fully explain how this area contributes in the cognition of magnitude in different dimensions.

It is speculated that if there is a shared brain area for perception of magnitudes in different dimensions, then they should interact with each other on the behavioural level. There are a few behavioural studies looking at the interference of these two dimensions. Xuan et al. [10] and Oliveri et al. [12] showed a temporal duration judgement can be biased by a number's magnitude. Droit-Volet et al [8] and Dormal [9] also showed the same effect. Importantly, they showed that duration judgement does not interfere with numerosity judgement.

We speculated that finding no interference of the temporal dimension on numerosity judgement reported in previous studies was confounded by the combination of the magnitudes used in the two dimensions. Numerosities used were all between 4 and 10, and durations used were between 1 and 8 seconds. We speculated that the combination of these magnitudes does not allow investigating the reciprocal interaction of numerosity and duration. Although it is known that quantities greater than 4 are processed using an analogue magnitude system (rather than subitizing), the discrete nature of numerosities (especially numerosities less than 10) prevents temporal processing to have any measurable interfering effect. By using increased numerosities and reduced durations, participants were forced to rely exclusively on the approximate magnitude system for both dimensions and subsequently the two-sided interaction of numerosity and duration became apparent, allowing us to investigate this interference reciprocally.

The results showed a bidirectional interference of numerosity and duration judgement: the more numerous a set was, the longer it was perceived and vice versa. Thus, our results show that numerosity and temporal magnitudes can interfere reciprocally. This might be because of common brain area(s). However, this does not fully reject the possibility of having some brain areas that are responsible for processing of magnitudes only in a few dimensions.

**Figure 6:**
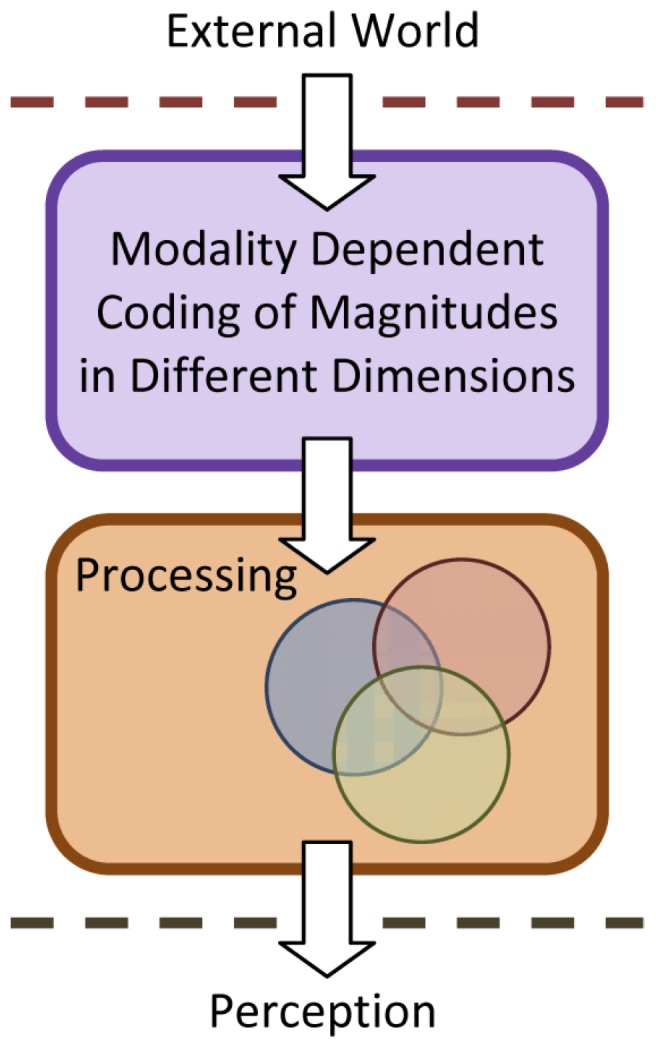
Mechanism of magnitude processing. This image highlights two points: different initial coding of information throughout the modalities and sharing of some brain areas among processing of magnitudes in two or more different dimensions (shown as circles).

In a more contradictory study, Agrillo, Ranpura and Butterworth [47], using auditory stimuli, showed that time and numerosity estimation are independent. Participants were asked to estimate the duration of the stimulus in one task, and the number of tones in another. They used durations between 5 and 13 seconds and numerosities between 11 and 19 tones. They did not find any interference effect of numerosity on duration or vice versa. Having the same task performed using visual stimuli, one expects to see at least interference of numerosity on duration estimation. We argue that perhaps the saliency of information and sensitivity to temporal and numerosity magnitudes in different modalities are different [11], Figure 6.

Judgement of the trials with *k*≤2 was difficult, as the ambiguity of the judgement was very high. As we expected, the performance of the participants for these trials was very poor. Therefore, major part of the analysis relies on trials with |*k*|>2. Although exclusion of trials with *k*≤2 could highly reduce the duration of the task, due to various reasons we did not remove these trials: (a) to have a clear s-shape psychometric function to better illustrate the effect, (b) to give participants the impression that the task consists of a continuum of easy and difficult trials, so that they wouldn't consider the inclusion of trials in which there is absolutely no difference between the two sets and (c) for the condition *k* = 0, we expected to achieve percentage response towards either of the sets at the chance level. Inclusion of this condition was necessary (i) to have a common baseline to be able to compare conditions *k* = ±4 with and (ii) to study bias towards either of the sets, and the results revealed that there was no bias.

Dimensions other than the total number of items could also affect the numerosity judgment, e.g. inter-item distances [48], overall luminance of the screen or a combination of these [49]. Therefore, it is not fully clear if the presented effect is an interference effect of total occupied area, or any other dimension, or a combination of these on duration processing in duration judgement task. Nevertheless, the main focus of this study was to investigate the interference of the temporal dimension on numerosity, which could not be confounded by any of possible confounding visual factors such as total occupied area.

Another limitation of the study is the possible contribution of iconic memory in judgement of numerosities due to no backward masking. This possibility, however, is highly unlikely as (a) the mean response times did not significantly change across different conditions, and (b) mean response times were too short to allow explicit counting from iconic memory. It should also be mentioned that this possibility could not interfere with the main focus of the experiment: interference of duration on numerosity.

In sum, the results of this study showed that, contrary to previous findings, the temporal dimension can interfere with numerosity. Although, the implications of the study might be limited to the durations and number of items that were used, it highlights the importance of correct selection of magnitudes in different dimensions in studies looking at processing of multiple dimensions in one study. Indeed, the mechanisms underlying the interference of different magnitudes continue to be poorly researched. A recent study by Matthews, Stewart and Wearden [50] on interaction of intensity and perception of duration suggests that it is the relative magnitude rather than absolute intensity that contributes in the interference of the two dimensions. In other words, it is the change in magnitudes that affects the target dimension, rather than the size of the magnitudes per se. Further research is necessary to gain a deeper understanding of these interactions.

To conclude, this study was able to identify a two-way interaction between time and numerosity judgement. These findings are in support of the vast body of literature suggesting magnitudes to be linked and interacting with each other, which might be due to their (partly-) shared location within the brain.

## References

[1] Stavy R, Tirosh D (2000). How students (mis-) understand science and mathematics: Intuitive rules.

[2] Levin I (1979). Interference of time-related and unrelated cues with duration comparisons of young children: Analysis of Piaget's formulation of the relation of time and speed.. Child Development.

[3] Levin I, Friedman WJ (1982). The nature and development of time concepts in children: The effects of interfering cues.. The developmental psychology of time.

[4] Levin I, Gilat I (1983). A developmental analysis of early time concepts: The equivalence and additivity of the effect of interfering cues on duration comparisons of young children.. Child Development.

[5] Walsh V (2003). A theory of magnitude: common cortical metrics of time, space and quantity.. Trends in Cognitive Sciences.

[6] Walsh V (2003). Time: the back-door of perception.. Trends in Cognitive Sciences.

[7] Bueti D, Walsh V (2009). The parietal cortex and the representation of time, space, number and other magnitudes.. Philos Trans R Soc Lond B Biol Sci.

[8] Droit-Volet S, Clément A, Fayol M (2003). Time and number discrimination in a bisection task with a sequence of stimuli: A developmental approach.. Journal of experimental child psychology.

[9] Dormal V, Seron X, Pesenti M (2006). Numerosity-duration interference: A Stroop experiment.. Acta Psychologica.

[10] Xuan B, Zhang D, He S, Chen X (2007). Larger stimuli are judged to last longer.. Journal of Vision.

[11] Roitman JD, Brannon EM, Andrews JR, Platt ML (2007). Nonverbal representation of time and number in adults.. Acta Psychologica.

[12] Oliveri M, Vicario C, Salerno S, Koch G, Turriziani P (2008). Perceiving numbers alters time perception.. Neuroscience letters.

[13] Cappelletti M, Freeman ED, Cipolotti L (2009). Dissociations and interactions between time, numerosity and space processing.. Neuropsychologia.

[14] Oyama T, Kikuchi T, Ichihara S (1981). Span of attention, backward masking, and reaction time.. Attention, Perception, & Psychophysics.

[15] Trick LM, Pylyshyn ZW (1994). Why are small and large numbers enumerated differently? A limited-capacity preattentive stage in vision.. Psychological Review.

[16] Mandler G, Shebo BJ (1982). Subitizing: An analysis of its component processes.. Journal of Experimental Psychology: General.

[17] Revkin SK, Piazza M, Izard V, Cohen L, Dehaene S (2008). Does Subitizing Reflect Numerical Estimation?. Psychological Science.

[18] Feigenson L, Dehaene S, Spelke E (2004). Core systems of number.. Trends in Cognitive Sciences.

[19] Dehaene S (2011). The number sense: How the mind creates mathematics.

[20] Oldfield R (1971). The assessment and analysis of handedness: the Edinburgh inventory.. Neuropsychologia.

[21] Brainard D (1997). The psychophysics toolbox.. Spatial vision.

[22] Pelli D (1997). The VideoToolbox software for visual psychophysics: Transforming numbers into movies.. Spatial vision.

[23] Dehaene S, Cohen L (1991). Two mental calculation systems: A case study of severe acalculia with preserved approximation.. Neuropsychologia.

[24] Lemer C, Dehaene S, Spelke E, Cohen L (2003). Approximate quantities and exact number words: Dissociable systems.. Neuropsychologia.

[25] Polk TA, Reed CL, Keenan JM, Hogarth P, Anderson CA (2001). A Dissociation between Symbolic Number Knowledge and Analogue Magnitude Information.. Brain and Cognition.

[26] Cipolotti L, Butterworth B, Denes G (1991). A specific deficit for numbers in a case of dense acalculia.. Brain.

[27] Piazza M, Izard V, Pinel P, Le Bihan D, Dehaene S (2004). Tuning curves for approximate numerosity in the human intraparietal sulcus.. Neuron.

[28] Piazza M, Mechelli A, Price CJ, Butterworth B (2006). Exact and approximate judgements of visual and auditory numerosity: An fMRI study.. Brain Research.

[29] Castelli F, Glaser DE, Butterworth B (2006). Discrete and analogue quantity processing in the parietal lobe: A functional MRI study.. Proc Natl Acad Sci U S A.

[30] Dormal V, Andres M, Dormal G, Pesenti M (2010). Mode-dependent and mode-independent representations of numerosity in the right intraparietal sulcus.. Neuroimage.

[31] Dormal V, Pesenti M (2009). Common and specific contributions of the intraparietal sulci to numerosity and length processing.. Human Brain Mapping.

[32] Dormal V, Andres M, Pesenti M (2008). Dissociation of numerosity and duration processing in the left intraparietal sulcus: a transcranial magnetic stimulation study.. Cortex.

[33] Cappelletti M, Barth H, Fregni F, Spelke ES, Pascual-Leone A (2007). rTMS over the intraparietal sulcus disrupts numerosity processing.. Experimental Brain Research.

[34] Harrington DL, Haaland KY, Knight RT (1998). Cortical networks underlying mechanisms of time perception.. The Journal of Neuroscience.

[35] Nichelli P, Alway D, Grafman J (1996). Perceptual timing in cerebellar degeneration.. Neuropsychologia.

[36] Nichelli P, Clark K, Hollnagel C, Grafman J (1995). Duration Processing after Frontal Lobe Lesionsa.. Annals of the New York Academy of Sciences.

[37] Coslett H, Shenton J, Dyer T, Wiener M (2009). Cognitive timing: Neuropsychology and anatomic basis.. Brain Research.

[38] Ferrandez A, Hugueville L, Lehericy S, Poline J, Marsault C (2003). Basal ganglia and supplementary motor area subtend duration perception: an fMRI study.. Neuroimage.

[39] Lewis PA, Miall RC (2003). Brain activation patterns during measurement of sub-and supra-second intervals.. Neuropsychologia.

[40] Pouthas V, George N, Poline JB, Pfeuty M, VandeMoorteele PF (2005). Neural network involved in time perception: an fMRI study comparing long and short interval estimation.. Human Brain Mapping.

[41] Belin P, McAdams S, Thivard L, Smith B, Savel S (2002). The neuroanatomical substrate of sound duration discrimination.. Neuropsychologia.

[42] Rao SM, Mayer AR, Harrington DL (2001). The evolution of brain activation during temporal processing.. Nature Neuroscience.

[43] Bueti D, Bahrami B, Walsh V (2008). Sensory and association cortex in time perception.. Journal of Cognitive Neuroscience.

[44] Alexander I, Cowey A, Walsh V (2005). The right parietal cortex and time perception: Back to Critchley and the Zeitraffer phenomenon.. Cognitive Neuropsychology.

[45] Lewis PA, Miall RC (2003). Distinct systems for automatic and cognitively controlled time measurement: evidence from neuroimaging.. Current opinion in neurobiology.

[46] Wiener M, Turkeltaub P, Coslett H (2010). The image of time: A voxel-wise meta-analysis.. Neuroimage.

[47] Agrillo C, Ranpura A, Butterworth B (2010). Time and numerosity estimation are independent: Behavioral evidence for two different systems using a conflict paradigm.. Cognitive Neuroscience.

[48] Allik J, Tuulmets T (1991). Occupancy model of perceived numerosity.. Perception & Psychophysics.

[49] Dehaene S, Izard V, Piazza M (2005). Control over non-numerical parameters in numerosity experiments.. http://www.unicog.org/docs/DocumentationDotsGeneration.doc.

[50] Matthews WJ, Stewart N, Wearden JH (2011). Stimulus intensity and the perception of duration.. Journal of Experimental Psychology: Human Perception and Performance.

